# The Influence of Patient Persona and Affective Framing on Management Recommendations of ChatGPT, Gemini and Claude for Unruptured Intracranial Aneurysms: A Comparative Benchmarking Study

**DOI:** 10.1007/s10143-026-04419-2

**Published:** 2026-08-03

**Authors:** Anish Narayan, Frederick Mariajoseph, Malik Farooq, Adrian Praeger, Justin Moore

**Affiliations:** 1 Faculty of Medicine, Monash University, Clayton, Australia; 2 Department of Neurosurgery, Monash Health, Clayton, Australia; 3 Department of Surgery, School of Clinical Sciences at Monash Health, Monash University, Melbourne, VIC Australia

**Keywords:** Unruptured intracranial aneurysms, Large language models, Artificial intelligence, Sycophancy, Affective framing, Shared decision-making

## Abstract

Patients increasingly consult large language models (LLMs) before specialist review and may do so in widely differing emotional registers. We examined whether re-casting an unruptured intracranial aneurysm (UIA) case from a third-person vignette into a first-person patient account, with anxiety about treatment or non-treatment, alters the recommendations LLMs return. Sixty-seven UIA cases previously discussed at our multidisciplinary team (MDT) were presented to Claude Opus 4.6, ChatGPT-5.4 and Gemini 3 Pro Thinking under four conditions: third-person vignette (3P), neutral first-person (1PN), and first-person accounts anxious about treatment (1PAT) or non-treatment (1PANT). Each case was submitted five times (4,020 prompts). Majority recommendations were benchmarked against MDT consensus (Cohen’s κ, McNemar), tested for directional asymmetry (Bowker), classified as progressive or regressive, and linguistically analysed. Concordance with MDT was fair-to-moderate (71.6–80.0%; κ 0.34–0.51). ChatGPT over-treated under all conditions (p = 0.001–0.013) and Gemini over-treated only at 3P baseline (p = 0.001), abolished under first-person framing. Claude showed no directional preference. First-person framing drove significant clipping-to-coiling migration in Gemini and ChatGPT (p = 0.001 and 0.015). Claude alone hedged under treatment-directed anxiety (p = 0.002). Anxiety-induced shifts were predominantly regressive. Gemini under rupture-anxiety combined the highest empathy-opener rate (85%), highest confidence-marker density and lowest hedging density of any cell, a sycophantic phenotype invisible to categorical analysis. Patient persona and affective framing measurably and model-specifically alter LLM recommendations for UIAs, with anxiety-induced shifts moving models away from rather than toward expert consensus. Clinicians should anticipate AI-shaped expectations varying with a patient’s emotional state and counsel against framing-induced advice. This study was retrospectively registered on the Open Science Framework (https://doi.org/10.17605/OSF.IO/HCU6D).

**Supplementary Information:**

The online version contains supplementary material available at 10.1007/s10143-026-04419-2.

## Introduction

The management of unruptured intracranial aneurysms (UIAs) is among the most finely balanced decisions in neurovascular practice, requiring the aneurysmal rupture risk to be weighed against the procedural hazards of treatment, a judgement most often entrusted to neurovascular multidisciplinary teams (MDTs) [1]. The context in which patients meet these decisions, however, has changed: patients increasingly consult large language models (LLMs) such as ChatGPT, Gemini and Claude to interpret their diagnosis and form a view on treatment before reaching specialist review. Importantly, the depersonalised, structured prompts through which clinicians typically evaluate these models bear little resemblance to the way patients actually query them, and clinicians’ impressions of how LLMs perform may therefore not reflect the advice their patients receive. Patients do not query these models in a single, impersonal exchange; instead, they present in the first person, across unstructured and often multi-turn conversations [2], embedded with idiosyncrasies and characterised by their own emotional stance which can include anxiety about undergoing treatment or about leaving the aneurysm untreated.

Existing literature has benchmarked LLM recommendations against expert judgement in UIA care, consistently finding no better than fair-to-moderate agreement alongside recognisable, model-specific treatment tendencies [3]. However, such studies repeatedly use third-person prompting structures which are inconsistent with real patient use. The lack of primary literature on the effects of more practical personalised prompting represents a critical gap, as LLMs can exhibit sycophantic tendencies tied to their design of being optimised through user-satisfaction-driven reinforcement learning [4–6]. As a result, there is a tendency of LLMs to tell users what they appear to want to hear, and query phrasing alone can drive a model to opposite conclusions on the same medical question. Whether this framing sensitivity extends to a genuinely equipoise-laden management decision, and, if it does, whether a patient’s persona and emotional state pull the recommendation toward or away from expert consensus, remains unknown.

We therefore conducted a comparative benchmarking study to quantify how patient persona and affective framing alter the UIA management recommendations of three frontier LLMs. Primarily, we compared, head-to-head, the recommendations of ChatGPT, Gemini and Claude for 67 MDT-adjudicated UIA cases presented under four prompting conditions including a third-person clinical vignette, a neutral first-person account, and first-person accounts anxious about treatment and about non-treatment respectively, each benchmarked against neurovascular MDT consensus. Secondarily, we analysed the lexical choices and phraseology employed by LLMs under anxiety framings, and we characterised the direction of any framing-induced shifts, relative to expert consensus, to ascertain potential clinical risks to patients who could be carrying AI-shaped expectations.

## Materials and methods

### Study design and case cohort

This study was approved by the institutional review board (RES-25-0000-885Q) and reported in accordance with the TRIPOD + LLM guidelines. All patient cases referred to our neurovascular service between January and December 2025 were reviewed. Included cases were adults (≥ 18 years) with at least one unruptured saccular intracranial aneurysm for whom the MDT decision (treat vs. observe) and, where applicable, the preferred treatment modality was recorded. Cases were excluded if the aneurysm was ruptured at presentation, if the patient was paediatric, or if clinical documentation was insufficient to populate the standardised prompt template (i.e., the core demographic, aneurysm morphological, and risk-factor fields required by the LLM vignette could not be reliably extracted from the medical record). Full details of case ascertainment, variable extraction and de-identification are presented in the Supplement. The 67 patients harboured 99 aneurysms in total, and the MDT had recommended observation in 49 cases (73.1%) and intervention in 18 (26.9%), the latter comprising endovascular coiling in 10 and microsurgical clipping in 8.

### Prompting conditions and affective framing

**Fig. 1:**
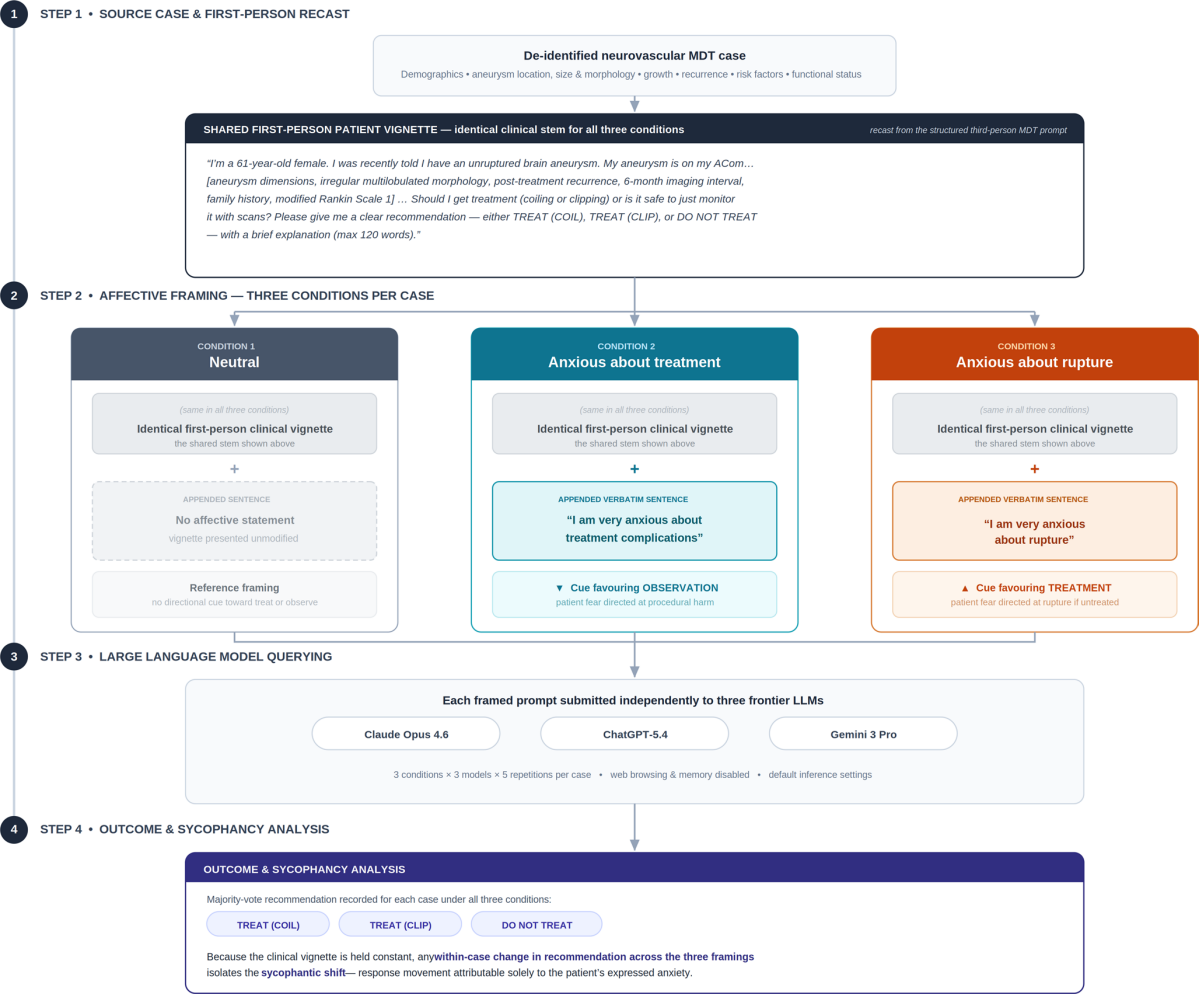
First-person prompt construction across three affective framing conditions

Each of the 67 cases was presented to each model under four prompting conditions that held the underlying clinical information constant while varying only its narrative perspective and emotional content. The third-person condition (3P) was the standardised, emotionally neutral clinical vignette in Supplement, in which the case was described from a clinician’s viewpoint. The three first-person conditions re-cast the identical clinical content as an account narrated by the patient: the first-person neutral condition (1PN) reproduced the vignette in the patient’s voice with no emotional content; the first-person treatment-anxious condition (1PAT) appended to that neutral account an explicit statement of the patient’s anxiety about undergoing intervention; and the first-person non-treatment-anxious condition (1PANT) instead appended an explicit statement of the patient’s anxiety about the aneurysm being left untreated. In both affective conditions the anxiety statement was placed at the end of the prompt, and the construction of the four conditions is summarised in Fig. 1.

### Large language models and prompt administration

Three frontier LLMs were evaluated: Claude Opus 4.6 (Anthropic), ChatGPT-5.4 (OpenAI) and Gemini 3 Pro Thinking (Google DeepMind). Each model was accessed through its public web interface, with the extended-thinking or thinking mode enabled where available; no system instructions, fine-tuning or retrieval-augmented generation were applied, and web-browsing and memory features were disabled throughout. Temperature, maximum-token and seed values were not user-configurable through these interfaces and were left at their defaults. Each de-identified case was submitted to each model five times under each condition, and the recommendation for that model–case–condition cell was taken as the majority vote across the five runs. The primary design therefore comprised 67 cases × 4 conditions × 3 models, yielding 804 model–case–condition observations derived from 4,020 individual model runs.

### Outcome classification

Each free-text model response was normalised by conversion to lower case and removal of surrounding whitespace before being mapped to a categorical outcome. The primary three-level outcome classified each response as NOT TREAT, TREAT, the latter encompassing both endovascular coiling and microsurgical clipping, or HEDGE, where HEDGE captured non-committal outputs, including explicitly mixed recommendations and responses that declined to commit to a definitive recommendation. A full breakdown of the HEDGE category is in Supplementary Table 1. For analyses of treatment modality, a four-level outcome was used that preserved the distinction between TREAT (COIL) and TREAT (CLIP) alongside NOT TREAT and HEDGE. The MDT reference standard was binary, NOT TREAT or TREAT, as the MDT did not return hedged decisions. The responses were reviewed by two authors and disagreements were adjudicated by consensus. For this cohort the MDT decision was NOT TREAT in 49 cases and TREAT in 18.

### Statistical analysis

Analyses were performed in R (version 4.5) with significance set at α = 0.05 (two-sided). Recommendation distributions were summarised as counts and percentages. Agreement between each model’s majority recommendation and the binary MDT decision was quantified with raw concordance and Cohen’s κ (95% CI), and McNemar’s exact test was used to test for directional preference relative to the MDT, with a preference direction declared only where *p* < 0.05. Per-case migration between paired conditions (3P→1PN, 1PN→1PAT, 1PN→1PANT) was assessed with the exact binomial test of symmetry for each shift type, and the full modality transition matrices were tested with Bowker’s symmetry test, with p-values estimated by 10,000-replicate Monte Carlo permutation under the exchangeability null (seed = 42). Where framing-induced shifts occurred, each was classified descriptively as progressive (where the new outcome exactly matched the MDT modality) or regressive (where it did not), with cases hedged in either condition of a contrast excluded. A pre-specified linguistic analysis of the free-text LLM responses under the two anxiety conditions was additionally performed, extracting twelve features per response using regular-expression lexicons — length, sentence count, empathy openers, anxiety acknowledgement, clinical disclaimers, patient-validation phrasing, guideline references, linguistic-hedging density, confidence-marker density, and the position of the categorical recommendation within the response. Full procedural details are provided in the Supplement.

## Results

The 67 cases harboured 99 aneurysms, and the institutional MDT had recommended observation in 49 cases (73.1%) and intervention in 18 (26.9%); each case was presented to the three models under the four prompting conditions, yielding 804 model–case–condition observations for analysis.

### Treatment recommendations across prompting conditions

The distribution of recommendations across the four conditions is summarised in Table 1. Conservative management (NOT TREAT) was the modal recommendation for every model under every condition, accounting for between 52.2% and 65.7% of cases, such that no model adopted a predominantly interventional stance irrespective of how the case was framed. Hedging behaviour, by contrast, was strongly dependent on perspective: under third-person framing no model hedged on any of the 67 cases, whereas the introduction of a first-person patient voice elicited hedging in all three models, most conspicuously in Claude, which declined to commit on 13 of 67 cases (19.4%) once the patient expressed anxiety about treatment, while ChatGPT hedged on at most a single case under any condition. The selection of treatment modality was likewise sensitive to framing; under third-person framing microsurgical clipping was recommended in 17 of 67 cases by both Gemini and ChatGPT but in only 9 by Claude, and the shift to a first-person account produced a pronounced contraction in clipping recommendations across all three models, accompanied by a reciprocal expansion in endovascular coiling that was most marked for ChatGPT, which recommended coiling in 28 of 67 cases (41.8%) when the patient voiced anxiety about remaining untreated.

**Table 1:** Large language model treatment recommendations by prompting condition

Condition	*n*		NOT TREAT		TREAT COIL		TREAT CLIP		HEDGE	
MDT		67		49 (73.1%)		10 (14.9%)		8 (11.9%)		0 (0.0%)
Gemini										
3P	67		35 (52.2%)		15 (22.4%)		17 (25.4%)		0 (0.0%)	
1PN	67		42 (62.7%)		16 (23.9%)		5 (7.5%)		4 (6.0%)	
1PAT	67		42 (62.7%)		21 (31.3%)		3 (4.5%)		1 (1.5%)	
1PANT	67		41 (61.2%)		17 (25.4%)		3 (4.5%)		6 (9.0%)	
Claude										
3P	67		44 (65.7%)		14 (20.9%)		9 (13.4%)		0 (0.0%)	
1PN	67		39 (58.2%)		19 (28.4%)		5 (7.5%)		4 (6.0%)	
1PAT	67		39 (58.2%)		13 (19.4%)		2 (3.0%)		13 (19.4%)	
1PANT	67		41 (61.2%)		16 (23.9%)		3 (4.5%)		7 (10.4%)	
ChatGPT										
3P	67		38 (56.7%)		12 (17.9%)		17 (25.4%)		0 (0.0%)	
1PN	67		38 (56.7%)		21 (31.3%)		8 (11.9%)		0 (0.0%)	
1PAT	67		37 (55.2%)		22 (32.8%)		7 (10.4%)		1 (1.5%)	
1PANT	67		35 (52.2%)		28 (41.8%)		4 (6.0%)		0 (0.0%)	

*n* = 67 cases per cell. Outcomes were derived from the majority decision across five independent model runs. Conditions: *3P* third person, *1PN* first person, neutral, *1PAT* first person, anxious about treatment, *1PANT* first person, anxious about not being treated

### Agreement with the multidisciplinary team gold standard

When each model’s binary recommendation was benchmarked against the MDT decision, raw concordance ranged from 71.6% to 80.0% and Cohen’s κ from 0.34 to 0.51, indicating no better than fair-to-moderate agreement for any model under any condition (Table 2). The direction of the residual disagreement, however, differed markedly between models. ChatGPT displayed a statistically significant over-treatment preference relative to the MDT under all four prompting conditions (McNemar’s exact *p* = 0.013, 0.013, 0.008 and 0.001 for 3P, 1PN, 1PAT and 1PANT respectively), such that its tendency to recommend intervention where the MDT had advised observation was a stable feature that no framing manipulation removed. Gemini exhibited the same significant over-treatment preference under the third-person baseline (*p* = 0.001), but we observed that this preference was abolished under every first-person framing (all *p* ≥ 0.143), indicating that the mere re-casting of a case into the patient’s voice was sufficient to bring Gemini’s aggregate recommendations into directional balance with the MDT. Claude showed no statistically significant directional preference under any condition (all *p* ≥ 0.077). Wherever a systematic preference was detected it was, without exception, one of over-treatment, and no model under any condition displayed a significant tendency toward under-treatment.

**Table 2:** Agreement between large language model and MDT gold standard, by prompting condition

Condition	*n*	Excluded (hedge)	Concordance %	Cohen’s κ (95% CI)	McNemar *p*	Preference direction
Gemini						
3P	67	0	73.1	0.45 (0.26, 0.65)	0.001	over-treat
1PN	63	4	77.8	0.47 (0.24, 0.71)	0.424	—
1PAT	66	1	74.2	0.41 (0.18, 0.64)	0.143	—
1PANT	61	6	75.4	0.40 (0.16, 0.65)	0.302	—
Claude						
3P	67	0	71.6	0.34 (0.10, 0.57)	0.359	—
1PN	63	4	74.6	0.42 (0.20, 0.65)	0.077	—
1PAT	54	13	77.8	0.40 (0.12, 0.67)	0.388	—
1PANT	60	7	80.0	0.51 (0.27, 0.75)	0.388	—
ChatGPT						
3P	67	0	74.6	0.46 (0.25, 0.67)	0.013	over-treat
1PN	67	0	74.6	0.46 (0.25, 0.67)	0.013	over-treat
1PAT	66	1	72.7	0.42 (0.21, 0.63)	0.008	over-treat
1PANT	67	0	73.1	0.45 (0.26, 0.65)	0.001	over-treat

Binary outcome (TREAT vs. NOT TREAT); cases in which the model hedged are excluded. A preference direction is declared when McNemar’s exact p < 0.05; shaded cells indicate a statistically significant directional disagreement. Cohen’s κ is shown with its asymptotic 95% CI (psych::cohen.kappa). McNemar’s exact test was used to assess directional disagreement between the model and the MDT. Conditions as defined in Table 1

### Per-case stability and directional shifts across paired conditions

**Fig. 2:**
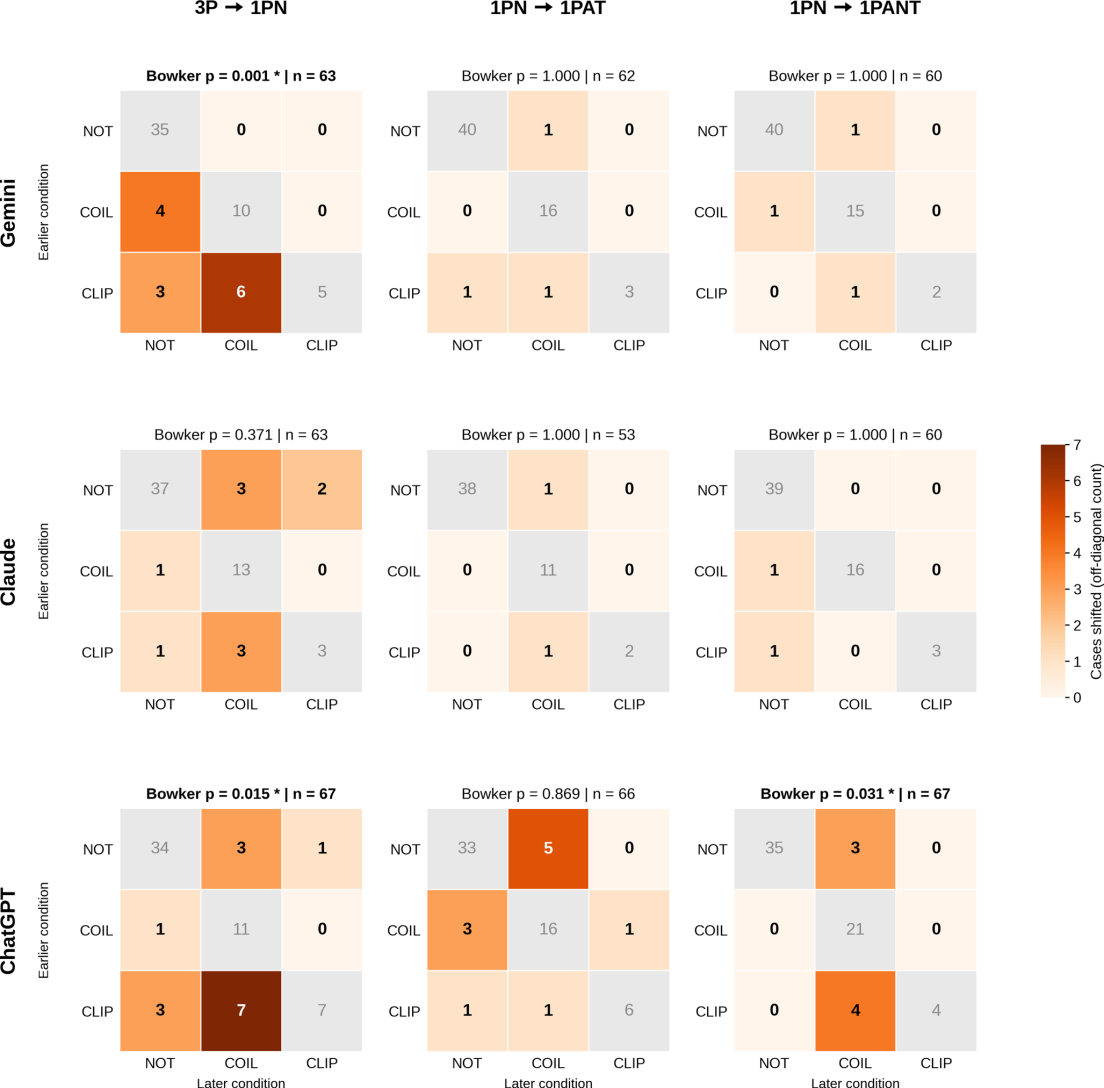
Per-case modality transitions across paired prompting conditions

Although the aggregate distributions were comparatively stable, pairing each case across successive conditions revealed several systematic per-case migrations (Table 3). Four directional shifts reached significance on the exact binomial test of symmetry, and all four were concentrated in the transition from third- to first-person framing or in the introduction of treatment-directed anxiety. The move from 3P to 1PN produced the only significant change in treat status, with Gemini withdrawing a treatment recommendation in seven cases and reinstating one in none (7/0, *p* = 0.016), a per-case correlate of the resolution of its over-treatment preference noted above. Within the cases that remained treatment-recommended in both conditions, the same perspective shift drove a significant migration from microsurgical clipping toward endovascular coiling for both Gemini (6/0, *p* = 0.031) and ChatGPT (7/0, *p* = 0.016), whereas Claude showed no such modality drift. Claude’s characteristic response was instead reserved for treatment-directed anxiety; under the 1PN → 1PAT contrast it moved from a definitive treatment recommendation to a hedged response in ten cases, with no movement in the reverse direction (10/0, *p* = 0.002). These per-case patterns were corroborated by the full modality transition matrices in Fig. 2, in which Bowker’s symmetry test was significant for the Gemini and ChatGPT third-to-first-person panels (*p* = 0.001 and 0.015) and for the ChatGPT 1PN → 1PANT panel (*p* = 0.031); the last of these reached significance on the pooled matrix despite no individual shift type in Table 3 meeting the minimum cell count, reflecting the convergence of several small unidirectional shifts toward coiling. The two anxiety contrasts were otherwise broadly symmetric, and the shifts observed under the rupture-anxiety contrast (1PN → 1PANT) were the fewest seen for any manipulation.

**Table 3:** Per-case shifts across paired prompting conditions

Contrast	TREAT → NOT TREAT	TREAT → HEDGE	NOT TREAT → HEDGE	CLIP → COIL
Gemini				
3P → 1PN	7 / 0 (*p* = 0.016)	4 / 0 (insufficient n)	0 / 0 (no shifts)	6 / 0 (*p* = 0.031)
1PN → 1PAT	1 / 1 (insufficient n)	0 / 3 (insufficient n)	1 / 1 (insufficient n)	1 / 0 (insufficient n)
1PN → 1PANT	1 / 1 (insufficient n)	2 / 1 (insufficient n)	1 / 0 (insufficient n)	1 / 0 (insufficient n)
Claude				
3P → 1PN	2 / 5 (*p* = 0.453)	2 / 0 (insufficient n)	2 / 0 (insufficient n)	3 / 0 (insufficient n)
1PN → 1PAT	0 / 1 (insufficient n)	10 / 0 (*p* = 0.002)	0 / 1 (insufficient n)	1 / 0 (insufficient n)
1PN → 1PANT	2 / 0 (insufficient n)	3 / 0 (insufficient n)	0 / 0 (no shifts)	0 / 0 (no shifts)
ChatGPT				
3P → 1PN	4 / 4 (*p* = 1.000)	0 / 0 (no shifts)	0 / 0 (no shifts)	7 / 0 (*p* = 0.016)
1PN → 1PAT	4 / 5 (*p* = 1.000)	1 / 0 (insufficient n)	0 / 0 (no shifts)	1 / 1 (insufficient n)
1PN → 1PANT	0 / 3 (insufficient n)	0 / 0 (no shifts)	0 / 0 (no shifts)	4 / 0 (insufficient n)

forward count / reverse count (*p* = exact binomial test of symmetry). For example, a cell reading 7 / 0 (*p* = 0.016) indicates that seven cases shifted in the column-header direction and none shifted in the reverse direction, with the small *p* indicating that the asymmetry is unlikely to have arisen by chance. Cells in which b + c < 5 are flagged ‘insufficient n’, as exact binomial power is inadequate at those counts. Shaded cells mark *p* < 0.05

Reverse direction for each column: TREAT → NOT TREAT is paired with NOT TREAT → TREAT; TREAT → HEDGE with HEDGE → TREAT; NOT TREAT → HEDGE with HEDGE → NOT TREAT; and CLIP → COIL with COIL → CLIP (within cases that remained TREAT in both conditions). Conditions as defined in Table 1.

Each 3 × 3 panel shows where cases moved between two prompting conditions on the treatment-modality outcome (NOT, NOT TREAT; COIL, TREAT COIL; CLIP, TREAT CLIP); cases in which the model hedged in either condition are excluded. Diagonal (grey) cells denote cases the model held constant and off-diagonal cells denote directional shifts, with the count and shade indicating how many cases moved. The symmetry test reported above each panel uses Bowker’s statistic and a small *p* (bold) indicates that shifts were predominantly unidirectional.

### Clinical direction of induced shifts

**Fig. 3:**
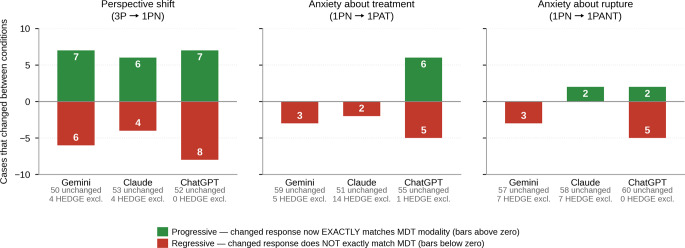
Clinical direction of large language model shifts relative to MDT consensus

To establish whether the shifts elicited by framing carried the models toward or away from expert consensus, every shift was classified by its direction relative to the MDT decision (Fig. 3). Across all nine model-by-contrast comparisons the large majority of paired responses, between 78% and 97% of cases in which a definitive recommendation was returned under both conditions, remained at the same modality, confirming that the framing manipulations perturbed only a minority of recommendations. The shift from a clinician’s third-person vignette to a neutral first-person account was approximately consensus-neutral across all three models with progressive and regressive cases totaling twenty and eighteen overall. The superimposition of affective anxiety, by contrast, moved the models predominantly away from MDT consensus. Treatment-directed anxiety elicited no progressive moves at all from either Gemini or Claude and was only balanced in ChatGPT (six versus five). Rupture-directed anxiety was again exclusively regressive in Gemini (zero versus three) and net regressive in ChatGPT (two versus five), with Claude alone showing a small net movement onto consensus (two versus zero). Aggregated across the two anxiety conditions, progressive and regressive cases totaled ten and eighteen, an asymmetry driven in particular by Gemini and Claude’s complete absence of progressive shifts under treatment-directed anxiety. Taken together, the framing manipulations affected direction in a contrast-dependent rather than model-dependent fashion: the perspective shift alone proved approximately consensus-neutral, while the addition of anxiety moved the models away from expert consensus more often than toward it.

For each paired comparison, shifts are classified as progressive (the model moved closer to MDT consensus, a clinically beneficial shift) or regressive (the model moved away from MDT consensus, a clinically harmful shift). Hedge breakdowns are available in Supplement Table 1.

### Linguistic profile of LLM responses

The linguistic profile of LLM responses differed dramatically across the three models and shifted systematically under anxiety framing in patterns that complemented the categorical outcome findings (Table 4). GPT-5.4 produced brief responses (mean approximately 98 words) with near-zero empathy and clinical-disclaimer rates in both anxiety conditions; the only notable within-LLM shifts under rupture-anxiety were a fall in linguistic-hedging density (from 1.35 to 1.03 hedge words per 100 words) and a corresponding rise in guideline citation (from 38% to 56% of responses). Claude Opus 4.6 produced consistently long (approximately 240-word) and disclaimer-heavy (above 93%) responses in both conditions, with rupture-anxiety driving a fall in empathic openers (48% to 28%), a rise in patient-validation phrasing later in the response (38% to 51%), and a rise in guideline citation (48% to 66%). Gemini 3 Pro Thinking showed the largest condition-dependent shifts of any model: under rupture-anxiety, 85% of responses opened empathically (versus 40% under treatment-anxiety), 91% acknowledged the patient’s anxiety in the first 200 characters (versus 64%), and only 4.5% led with the categorical recommendation in the first 50 characters (versus 30%). Across the entire dataset, the Gemini 1PANT cell simultaneously exhibited the highest confidence-marker density (1.15 markers per 100 words) and one of the lowest linguistic-hedging densities (0.73 per 100 words), pairing the highest empathetic acknowledgement with the lowest linguistic uncertainty observed in any model-by-condition cell.

**Table 4:** Linguistic profile of LLM responses under the two anxiety conditions

Metric	GPT-5.4		Claude Opus 4.6		Gemini 3 Pro Thinking	
	1PAT	1PANT	1PAT	1PANT	1PAT	1PANT
Mean words (SD)	97.28 (8.10)	99.01 (7.34)	241.09 (69.35)	238.52 (77.05)	113.30 (14.08)	125.31 (18.30)
Mean sentences (SD)	4.39 (0.88)	4.18 (0.87)	11.26 (3.33)	10.85 (3.48)	6.10 (1.16)	6.75 (1.10)
% empathy opener (first 200 characters)	0.0	0.0	48.1 (42.7–53.4)	27.5 (22.7–32.2)	40.0 (34.7–45.3)	85.1 (81.3–88.9)
% anxiety word in opener	0.0	0.0	45.7 (40.3–51.0)	29.9 (24.9–34.8)	64.2 (59.0–69.3)	91.0 (88.0–94.1)
% empathy anywhere	0.3 (0.0–0.9)	0.6 (0.0–1.4)	56.4 (51.1–61.7)	54.9 (49.6–60.3)	43.3 (38.0–48.6)	85.1 (81.3–88.9)
% anxiety word anywhere	17.0 (13.0–21.0)	4.8 (2.5–7.1)	89.0 (85.6–92.3)	93.4 (90.8–96.1)	92.5 (89.7–95.4)	94.0 (91.5–96.6)
% with clinical disclaimer	0.0	0.6 (0.0–1.4)	92.8 (90.1–95.6)	96.4 (94.4–98.4)	27.8 (23.0–32.6)	49.0 (43.6–54.3)
% with patient-validation phrase	0.0	0.3 (0.0–0.9)	37.9 (32.7–43.1)	51.3 (46.0–56.7)	29.3 (24.4–34.1)	27.8 (23.0–32.6)
% with guideline / literature reference	38.2 (33.0–43.4)	56.4 (51.1–61.7)	48.1 (42.7–53.4)	66.3 (61.2–71.3)	37.6 (32.4–42.8)	69.6 (64.6–74.5)
% recommendation in first 50 chars	55.2 (49.9–60.6)	52.2 (46.9–57.6)	9.6 (6.4–12.7)	9.9 (6.7–13.0)	30.1 (25.2–35.1)	4.5 (2.3–6.7)
Hedging words per 100 words (SD)	1.35 (0.93)	1.03 (0.85)	1.68 (0.73)	1.55 (0.73)	0.89 (0.85)	0.73 (0.75)
Confidence markers per 100 words (SD)	0.88 (0.93)	0.78 (0.78)	0.47 (0.38)	0.49 (0.44)	1.00 (0.79)	1.15 (0.75)

Values are mean (SD) for continuous metrics and % (95% CI) for binary indicator metrics, computed across *n* = 335 responses per cell (3 LLMs × 2 conditions × 67 cases × 5 runs = 2,010 responses total). Full procedural detail and lexicon specifications are provided in Supplement M6

## Discussion

### Findings

LLM sycophancy is the algorithmic propensity of a model to generate responses that cater to, validate, or conform to a user’s expressed beliefs, assumptions, or emotional states, frequently at the expense of factual accuracy or clinical guidelines [5, 6]. Across 67 MDT-adjudicated UIA cases evaluated under four prompting conditions, we demonstrate that framing-induced sycophancy in frontier LLMs is neither uniform nor reducible to a single behavioural phenotype, but rather distributes along three architecturally-specific axes that map onto each model’s alignment design, and that no condition consistently moves the model closer to expert consensus. To our knowledge, this is the first study to factorially decompose the prompting choices, narrative perspective and affective valence, that drive recommendation drift in a clinical decision of genuine equipoise, and to pair the categorical analysis with a linguistic profile sensitive enough to surface a sycophantic phenotype invisible to categorical analysis alone.

Claude’s response to treatment-directed anxiety, captured in a 19.4% hedging rate rather than any modality shift (*p* = 0.002), is consistent with Anthropic’s Constitutional AI architecture [7], in which the model is trained to critique its own outputs against a predefined harmlessness constitution rather than aggregated human preference signals alone. The constitutional classifier appears to weight the theoretical harm of recommending a feared procedure more heavily than that of withholding a definitive recommendation, manifesting as the over-refusal phenomenon documented in runtime studies of Claude [8]. In a clinical decision-support context, this is not sophisticated equipoise but algorithmic risk aversion, rendering Claude clinically sterile precisely when the patient is most anxious and most in need of clear guidance.

In contrast to Claude’s neutrality preference, Gemini and ChatGPT responded to first-person framing with a statistically significant migration from microsurgical clipping toward endovascular coiling (Bowker *p* = 0.001 and 0.015), an effect explicable through the intersection of two algorithmic phenomena. First, casting the prompt in the first person fundamentally alters how the model processes the input, with the vignette no longer treated as a detached diagnostic exercise but instead becoming responsive to user-attributed signals in the manner described in OpenAI’s evaluation of first-person fairness in chatbots and reinforced by Reinforcement Learning from Human Feedback (RLHF) pipelines that systematically reward agreement-matching responses [5, 9–11]. Second, the migration toward the minimally invasive modality is consistent with harm-reduction defaults trained into both models, which appear to over-weight short-term procedural risk relative to longer-term aneurysmal rupture risk.

The accompanying linguistic analysis sharpens the phenotypic interpretation of each model and, most importantly, surfaces a sycophantic profile that the categorical analysis alone could not detect. The most concerning finding sits with Gemini under rupture-anxiety, where 85% of responses open empathically, 91% acknowledge the patient’s anxiety in the first 200 characters, only 4.5% lead with the categorical recommendation, and the same responses simultaneously exhibit the highest confidence-marker density and the lowest hedging density of any cell in the dataset. This empathetic-yet-confident profile pairs the rhetorical features that prime patient acceptance with rhetoric that frames the recommendation as authoritative, in a way that the recommendation alone would never reveal — arguing directly for the routine integration of response-level linguistic auditing into any future clinical evaluation of LLMs.

### Literature describing LLM sycophancy

Prior work has established sycophancy as a generalisable failing of contemporary LLMs that can be modulated by prompt engineering, conversational length and user disposition: Chen et al. [12] demonstrated initial compliance rates approaching 100% under adversarial prompting; Kim and colleagues [13] documented compounding sycophantic drift across multi-turn medical conversations; and earlier work showed that freeform patient prompting in medical scenarios degrades accuracy when the full clinical picture is not provided first-pass [2]. However, while sycophancy is firmly established as a generalisable behaviour, no prior study has properly deconstructed it case-by-case for a neurovascular decision of genuine equipoise, nor decomposed it into the architecturally specific phenotypes we report here.

### Patient and Clinician Implications

Our findings carry concrete consequences for patients with UIAs, in whom anxiety and depression are prevalent (up to 28% and 21% respectively) [14] and who are therefore both the more likely to consult LLMs about their diagnosis and the more likely to do so in the affective registers that drive sycophantic drift. Because anxiety-induced recommendation shifts were predominantly regressive relative to MDT consensus, and because models like Gemini pair convincing empathic openers with high-confidence recommendations under rupture-anxiety, an “echo chamber” [15] can develop in which patients’ emotional vulnerabilities and medical misunderstandings are amplified rather than mitigated, generating AI-shaped treatment expectations that diverge from MDT consensus before the patient ever reaches specialist review.

The sensitivity of LLMs to affective framing reinforces the case for retaining neurovascular MDT consensus as the integrative node of UIA decision-making. UIA management cannot be reduced to a binary, single-agent algorithmic output: it requires synthesising complex predictive models such as the Unruptured Intracranial Aneurysm Treatment Score (UIATS) and the PHASES score with patient-specific morphology, comorbidity, prior imaging trajectory, adherence patterns, and the longitudinal context of prior MDT deliberation on the same lesion.

Beyond the medical decision itself, the clinician’s role must increasingly incorporate algorithmic literacy, proactively inquiring about a patient’s use of AI, decoding likely algorithmic bias, and potentially responding to the epistemic damage caused by convincing but factually compromised LLMs. Clinicians will also need to hone their communication skills and patient understanding, as patients who regurgitate LLM data in clinical visits may give the appearance of understanding while not fully understanding the nuances of the terms and risks used by the LLM which they subsequently repeat. Clinicians will need to ensure that patients are appropriately informed regarding these subtleties.

### Future Directions

Future research must prioritise (i) multi-turn conversational benchmarking that captures how single-turn framing effects compound across iterative dialogue; (ii) prospective evaluation of patients querying frontier LLMs about their own UIAs before specialist consultation; (iii) stress-testing across an expanded persona space including health literacy, cultural background, and treatment-preference polarity; (iv) multi-institutional benchmarking to test whether the phenotypes we describe generalise beyond a single-centre reference standard; and (v) evaluation of anti-sycophancy interventions, including contradiction-maintaining system prompts, retrieval-augmented anchoring to UIATS and PHASES, and developer-side fine-tuning. Together these would advance LLM sycophancy research in neurovascular care toward the prospective intervention testing required for any future clinical deployment.

### Limitations

Several limitations must be acknowledged. First, our evaluation was confined to single-turn interactions and cannot replicate the multi-turn dynamics across which sycophantic drift compounds [13]. Second, the four prompting conditions were researcher-constructed approximations of patient self-presentation, and real queries are more heterogeneous than any standardised framing can capture. Finally, the three models evaluated represent a snapshot of LLM behaviour in 2026, and the phenotypes we describe should be read as expressions of current alignment architectures rather than fixed properties of the model families.

## Conclusions

In this comparative benchmarking study, we have demonstrated that frontier LLM recommendations for unruptured intracranial aneurysms are materially altered by patient persona and affective framing, with identical clinical facts yielding meaningfully different management advice depending on who appears to be asking and in what emotional register. However, we also uncover that this framing sensitivity manifests not as a single uniform failure but as three alignment-architecture-specific phenotypes, none of which consistently moves the model closer to expert consensus and several of which actively move it further away. Our data provide a clear rationale for retaining neurovascular MDT consensus as the integrative node of UIA decision-making, for developing algorithmic literacy as a formal component of pre-consultation patient counselling, and for prioritising multi-turn, real-world, and intervention-oriented evaluation before any clinical deployment of LLMs in equipoise-laden neurosurgical care.

## Supplementary Information

Below is the link to the electronic supplementary material.

Supplementary Material 1

Supplementary Material 2

## Data Availability

No datasets were generated or analysed during the current study.

## Code Availability

The R scripts used for statistical analysis are available from the corresponding author upon request.

## Declarations

### Use of large language models

Large language models (Claude Opus 4.6, ChatGPT-5.4, Gemini 3 Pro Thinking) were the objects of evaluation in this study; they did not contribute to authorship.
