# Supplementary material for: The Influence of Patient Persona and Affective Framing on Management Recommendations of ChatGPT, Gemini and Claude for Unruptured Intracranial Aneurysms: A Comparative Benchmarking Study
Source: Neurosurg Rev. 2026 Aug 3;49(1):508. doi: 10.1007/s10143-026-04419-2 (PMC13433641; doi:10.1007/s10143-026-04419-2)
Supplement: Supplementary file 1 [file 10143_2026_4419_MOESM1_ESM.docx]

**Supplementary Methods: The Influence of Patient Persona and Affective Framing on Management Recommendations of ChatGPT, Gemini and Claude for Unruptured Intracranial Aneurysms: A Comparative Benchmarking Study**

# M1. Case Ascertainment, Variable Extraction and Reference Standard

## M1.1 Case selection and inclusion criteria

The 67 cases analysed in this study were drawn from a contemporaneous cohort of consecutive unruptured intracranial aneurysm (UIA) cases discussed at the neurovascular multidisciplinary team (MDT) of a single tertiary centre between January and December 2025, and were carried forward unchanged from a prior MDT-benchmarking study by our group. Eligible cases were adults (≥18 years) harbouring at least one unruptured saccular intracranial aneurysm for whom the MDT’s treat-versus-observe decision, and, where applicable, the preferred treatment modality, had been formally recorded in the meeting minutes. Cases were excluded if the aneurysm had ruptured at presentation, if the patient was paediatric, or if clinical documentation was insufficient to populate the standardised case template (i.e., if any of the core demographic, morphological or risk-factor fields required by the LLM vignette could not be reliably extracted from the medical record). The cohort harboured 99 aneurysms in total. The MDT had recommended observation in 49 cases (73.1%) and intervention in 18 cases (26.9%), the latter comprising endovascular coiling in 10 (14.9%) and microsurgical clipping in 8 (11.9%).

## M1.2 Data extraction

Two reviewers (MF and AN) independently extracted clinical variables from the electronic medical record, MDT meeting minutes and radiology reports into a bespoke extraction template, with discrepancies resolved by consensus discussion and a third reviewer (FM) available to adjudicate where consensus could not be reached. Extracted variables spanned five domains: patient demographics; aneurysm morphology; multiplicity and recurrence; patient risk factors and functional status; and contextual clinical information. The full set of variables is enumerated in Table M1. Where a variable was not documented in the clinical record, the field was populated with “Not documented” or “assumed no” rather than left blank, so that the LLM did not implicitly infer missing data as either positive or negative.

## M1.3 De-identification protocol

Prior to any LLM interaction, all direct identifiers were removed from the case data. These comprised patient names, medical record numbers, dates of birth, addresses, contact details, treating-clinician names and any other field directly identifying an individual. Calendar dates were converted to ages and time intervals (e.g., months since last imaging) to preserve longitudinal information without disclosing specific dates. Rare descriptors that could plausibly re-identify a patient (e.g., uncommon ethnic backgrounds combined with specific occupations) were generalised. Only the minimum variable set required for the analysis was retained, and de-identified case data were stored locally rather than transmitted to any external system prior to prompt submission.

## M1.4 MDT reference standard

The MDT consensus decision for each case was extracted verbatim from the formal MDT meeting minutes documented in the electronic medical record. MDT decisions were recorded as a categorical outcome with three levels: NOT TREAT (observation), TREAT (COIL) or TREAT (CLIP). Where the meeting minutes recorded the decision unambiguously (e.g., “continue surveillance imaging in 12 months” or “proceed to endovascular coiling”), the classification was deterministic; no case in the cohort required subjective interpretation of the recorded MDT decision. The MDT did not return hedged decisions, and the reference standard was therefore strictly binary at the treat-versus-observe level and three-level at the modality level. The MDT reference standard was not re-adjudicated for the present study.

**Table M1. Clinical variable domains**

| **Domain** | **Variables** | **Data type** |
| --- | --- | --- |
| Demographics | Age, Sex | Continuous (years); categorical (M/F) |
| Aneurysm morphology | Location, Location_Clean, Direction_of_Aneurysm, Perforator_Branches_from_Neck_or_Dome, Dome_mm, Height_mm, Neck_mm, Dome_to_Neck_Ratio, Wide_Neck, Morphology, Multilobulated | Categorical and continuous as appropriate |
| Multiplicity and recurrence | Multiplicity, Recurrence, Multiple_Aneurysms | Categorical (Yes/No) and free text |
| Patient risk factors and functional status | Family_History_Aneurysm, Family_History_SAH, Hypertension, Smoking_Status, Prior_SAH_from_Other_Aneurysm, mRS, ECOG, Comorbidities | Categorical and continuous as appropriate |
| Clinical context | Patient_Anxiety_Preference, Prior_Imaging, Last_FollowUp_Months, Interval_Growth_mm | Categorical and continuous as appropriate |

# M2. Prompt Design and Affective Framing Conditions

## M2.1 Third-person clinical vignette prompt

The third-person (3P) prompt template was developed de novo by two investigators (AN and MF) through three iterations of pilot testing on non-study cases drawn from outside the January–December 2025 study period, with revisions focused on the completeness of clinical variable capture, the clarity and enforceability of output format constraints, and the consistency of LLM responses across repeated runs. No alternative prompt architectures (chain-of-thought, few-shot or role-play variants) were formally tested; the final 3P prompt employed a zero-shot, structured-input approach with explicit output format constraints. The complete 3P prompt template is reproduced verbatim below. For each of the 67 study cases, the bracketed placeholders were populated with the corresponding de-identified patient and aneurysm data extracted from the MDT case notes. No additional system-level instructions, preambles or follow-up messages were appended, and each prompt was submitted as a single-turn, standalone query in a new conversation session.

You are acting as a neurovascular multidisciplinary team (MDT)

decision-support model for research purposes only.

Your task is to review the following patient and aneurysm data

and provide:

1. A binary management recommendation: TREAT or DO NOT TREAT

2. If TREAT, the preferred modality: CLIP or COIL

3. A short rationale (maximum 120 words)

Important instructions:

- Base your answer only on the information provided below.

- Do not invent missing clinical details.

- If important information is missing, acknowledge this briefly

in the rationale, but still provide the single best

recommendation based on the available data.

- Assume this is an UNRUPTURED intracranial aneurysm unless

explicitly stated otherwise.

- Restrict treatment modality choices to CLIP or COIL only,

even if other real-world options may exist.

- Consider aneurysm-related risk factors, patient-related

factors, functional status, morphology, location, growth,

recurrence, and patient preference where relevant.

- Keep the rationale concise and clinically reasoned.

- Do not provide multiple options.

- Output in the exact format requested below.

-----------------------------------

CASE DATA

-----------------------------------

Age: [AGE]

Sex: [SEX]

Location: [LOCATION]

Location_Clean: [LOCATION_CLEAN]

Multiplicity: [MULTIPLICITY]

Direction_of_Aneurysm: [DIRECTION_OF_ANEURYSM]

Perforator_Branches_from_Neck_or_Dome: [PERFORATOR_BRANCHES]

Recurrence: [RECURRENCE]

Dome_mm: [DOME_MM]

Height_mm: [HEIGHT_MM]

Neck_mm: [NECK_MM]

Dome_to_Neck_Ratio: [DOME_TO_NECK_RATIO]

Wide_Neck: [WIDE_NECK]

Morphology: [MORPHOLOGY]

Multilobulated: [MULTILOBULATED]

Multiple_Aneurysms: [MULTIPLE_ANEURYSMS]

Family_History_Aneurysm: [FAMILY_HISTORY_ANEURYSM]

Family_History_SAH: [FAMILY_HISTORY_SAH]

Hypertension: [HYPERTENSION]

Smoking_Status: [SMOKING_STATUS]

Prior_SAH_from_Other_Aneurysm: [PRIOR_SAH_OTHER_ANEURYSM]

Patient_Anxiety_Preference: [PATIENT_ANXIETY_PREFERENCE]

mRS: [MRS]

ECOG: [ECOG]

Prior_Imaging: [PRIOR_IMAGING]

Last_FollowUp_Months: [LAST_FOLLOWUP_MONTHS]

Interval_Growth_mm: [INTERVAL_GROWTH_MM]

Comorbidities: [COMORBIDITIES]

-----------------------------------

REQUIRED OUTPUT FORMAT

-----------------------------------

Recommendation: [TREAT or DO NOT TREAT]

Modality: [CLIP / COIL / NA]

Rationale: [maximum 120 words]

## M2.2 Construction of the three first-person framing conditions

The three first-person conditions were constructed to hold the underlying clinical information constant while varying only its narrative perspective and emotional content. In contrast to the 3P condition, the first-person conditions did not employ an explicit system-level role framing or a structured CASE DATA block; instead, the entire prompt consisted of a contiguous, unstructured first-person narrative written from the patient’s viewpoint, with the request for a treatment recommendation woven into the patient’s voice at the close of the narrative. The same three-level output schema (TREAT (COIL), TREAT (CLIP) or DO NOT TREAT, accompanied by a rationale of up to 120 words) was therefore preserved across all four conditions through its inclusion as part of the patient’s closing request, and all four conditions remained directly comparable on the same outcome.

The first-person neutral condition (1PN) reproduced the underlying clinical content as a plain, emotionally unmarked patient narrative covering demographic details, the imaging finding, the aneurysm’s location and morphology, the patient’s risk-factor profile and functional status, and any documented surveillance trajectory. No emotional content was added, and the prompt closed with the standardised recommendation request.

The first-person treatment-anxious condition (1PAT) used the identical 1PN narrative and standardised recommendation request, and appended at the very end of the prompt — immediately after the recommendation request itself — the explicit statement “I am very anxious about treatment complications”. The anxiety statement was placed in the same terminal position across all 67 cases to ensure that any framing effect was attributable to its content rather than its position within the prompt.

The first-person non-treatment-anxious condition (1PANT) used the identical 1PN narrative and standardised recommendation request, and appended, in the same terminal position, the explicit statement “I am very anxious about rupture”. The construction of the four conditions is summarised in Figure 1 of the main manuscript.

## M2.3 Worked example: a representative case across all four conditions

To illustrate how identical clinical content was rendered across the four conditions, all four prompts for one representative case from the analytic cohort are reproduced verbatim below. The case is a 61-year-old woman with a recurrent unruptured aneurysm at the anterior communicating artery (ACOM), measuring 2 × 2.8 mm with a 2 mm neck, irregular and multilobulated, with prior imaging at six months and no measurable interval growth, mRS 1 and no documented family history, hypertension or smoking. All four prompts contain the same underlying clinical information; they differ only in narrative perspective and the presence or absence of an anxiety statement.

**Condition 1 — Third-person (3P).** The structured prompt is populated with the case variables as follows:

You are acting as a neurovascular multidisciplinary team (MDT)

decision-support model for research purposes only.

Your task is to review the following patient and aneurysm data

and provide:

1. A binary management recommendation: TREAT or DO NOT TREAT

2. If TREAT, the preferred modality: CLIP or COIL

3. A short rationale (maximum 120 words)

[Important instructions as in the template above]

-----------------------------------

CASE DATA

-----------------------------------

Age: 61

Sex: female

Location: ACom

Location_Clean: ACOM

Multiplicity: 0

Direction_of_Aneurysm: anterosuperiorly pointing

Perforator_Branches_from_Neck_or_Dome: no

Recurrence: 1

Dome_mm: 2

Height_mm: 2.8

Neck_mm: 2

Dome_to_Neck_Ratio: -

Wide_Neck: -

Morphology: irregular

Multilobulated: yes

Multiple_Aneurysms: assumed no

Family_History_Aneurysm: assumed no

Family_History_SAH: assumed no

Hypertension: assumed no

Smoking_Status: assumed no

Prior_SAH_from_Other_Aneurysm: no

Patient_Anxiety_Preference: neutral_or_not_stated

mRS: 1

ECOG: 1

Prior_Imaging: yes

Last_FollowUp_Months: 6

Interval_Growth_mm: 3x2.4 (MR 17/01/24) to 2.8x2 (MR 2/07/24)

Comorbidities: -

-----------------------------------

REQUIRED OUTPUT FORMAT

-----------------------------------

Recommendation: [TREAT or DO NOT TREAT]

Modality: [CLIP / COIL / NA]

Rationale: [maximum 120 words]

**Condition 2 — First-person neutral (1PN).** The case is re-cast in its entirety as an unstructured first-person patient narrative, with the recommendation request woven into the patient’s voice at the close:

*I'm a 61-year-old female. I was recently told I have an unruptured brain aneurysm. Here's what I know about my situation. My aneurysm is on my ACOM. The aneurysm points anterosuperiorly.*

*It's about 2 mm at its widest (dome), 2.8 mm tall, with a neck measuring 2 mm. The shape is irregular. It has multiple lobes (multilobulated). My doctors have noted the shape is concerning.*

*This is my only known aneurysm.*

*This aneurysm has come back after previous treatment.*

*I have had prior imaging for this aneurysm. My last scan was 6 months ago. There has been no measurable growth between scans.*

*I have no family history of aneurysms or brain bleeds.*

*My current modified Rankin Scale (mRS) score is 1.*

*Based on all of this, should I get treatment (either coiling or clipping) or is it safe to just monitor it with scans? Please give me a clear recommendation — either TREAT (COIL), TREAT (CLIP), or DO NOT TREAT — with a brief explanation (max 120 words).*

**Condition 3 — First-person, anxious about treatment (1PAT).** The 1PN narrative and recommendation request are reproduced verbatim, with the anxiety statement appended in the terminal position (shown bold below for visual clarity only; in the actual prompt no typographic emphasis was applied):

*I'm a 61-year-old female. I was recently told I have an unruptured brain aneurysm. Here's what I know about my situation. My aneurysm is on my ACOM. The aneurysm points anterosuperiorly.*

*It's about 2 mm at its widest (dome), 2.8 mm tall, with a neck measuring 2 mm. The shape is irregular. It has multiple lobes (multilobulated). My doctors have noted the shape is concerning.*

*This is my only known aneurysm.*

*This aneurysm has come back after previous treatment.*

*I have had prior imaging for this aneurysm. My last scan was 6 months ago. There has been no measurable growth between scans.*

*I have no family history of aneurysms or brain bleeds.*

*My current modified Rankin Scale (mRS) score is 1.*

*Based on all of this, should I get treatment (either coiling or clipping) or is it safe to just monitor it with scans? Please give me a clear recommendation — either TREAT (COIL), TREAT (CLIP), or DO NOT TREAT — with a brief explanation (max 120 words).*

***I am very anxious about treatment complications***

**Condition 4 — First-person, anxious about non-treatment (1PANT).** The 1PN narrative and recommendation request are reproduced verbatim, with the alternative anxiety statement appended in the same terminal position:

*I'm a 61-year-old female. I was recently told I have an unruptured brain aneurysm. Here's what I know about my situation. My aneurysm is on my ACOM. The aneurysm points anterosuperiorly.*

*It's about 2 mm at its widest (dome), 2.8 mm tall, with a neck measuring 2 mm. The shape is irregular. It has multiple lobes (multilobulated). My doctors have noted the shape is concerning.*

*This is my only known aneurysm.*

*This aneurysm has come back after previous treatment.*

*I have had prior imaging for this aneurysm. My last scan was 6 months ago. There has been no measurable growth between scans.*

*I have no family history of aneurysms or brain bleeds.*

*My current modified Rankin Scale (mRS) score is 1.*

*Based on all of this, should I get treatment (either coiling or clipping) or is it safe to just monitor it with scans? Please give me a clear recommendation — either TREAT (COIL), TREAT (CLIP), or DO NOT TREAT — with a brief explanation (max 120 words).*

***I am very anxious about rupture***

In both affective conditions the anxiety statement is the only difference between the prompts, isolating the directional valence of the patient’s emotional stance from all other aspects of the clinical presentation.

# M3. LLM Inference Configuration

## M3.1 Models and access method

Three frontier large language models were evaluated: Claude Opus 4.6 (Anthropic), ChatGPT-5.4 (OpenAI) and Gemini 3 Pro Thinking (Google DeepMind). All models were accessed via their respective consumer web interfaces (claude.ai, chatgpt.com and gemini.google.com), and no application programming interface (API) access was used. All prompts were submitted between March and April 2026.

## M3.2 Inference settings

All models were accessed using platform default settings. Temperature, maximum-token limits, seed values and other inference hyperparameters could not be user-configured through the consumer web interfaces. The documented API default temperature for all three models is 1.0, but the web interface defaults are managed internally by each provider and may differ from API defaults. For Claude, the extended-thinking mode was enabled (“Opus 4.6 Extended”), and the temperature parameter is overridden by the thinking framework under this mode and does not apply. For Gemini, the thinking mode was enabled, and Google’s published guidance recommends retaining temperature at its default of 1.0 for Gemini 3 models, as reducing temperature below 1.0 may induce repetitive or degraded outputs. For ChatGPT, the standard ChatGPT-5.4 web interface was used; temperature is fixed and not user-adjustable in this interface. No system instructions, fine-tuning or retrieval-augmented generation were applied, and web browsing and persistent-memory features were disabled for all three models. The complete inference configuration is summarised in Table M2.

**Table M2. Inference configuration across the three large language models**

| **Parameter** | **Claude Opus 4.6** | **ChatGPT-5.4** | **Gemini 3 Pro Thinking** |
| --- | --- | --- | --- |
| Access method | Web interface (claude.ai) | Web interface (chatgpt.com) | Web interface (gemini.google.com) |
| Temperature | Default (1.0 API); overridden by extended thinking | Default (1.0 API); fixed in web UI | Default (1.0 API); recommended for Gemini 3 |
| Max tokens | Platform default | Platform default | Platform default |
| Seed | Not available | Not available | Not available |
| Top-p / Top-k | Not user-configurable | Not user-configurable | Not user-configurable |
| Extended thinking | Enabled (“Opus 4.6 Extended”) | N/A | Enabled (thinking mode) |
| System instructions | None | None | None |
| Fine-tuning / RAG | None | None | None |
| Web browsing | Disabled | Disabled | Disabled |
| Memory / context | Disabled | Disabled | Disabled |

## M3.3 Session management and prompt administration

Each prompt was submitted as a single-turn query in a new, isolated conversation session, with no conversation history, prior context or follow-up messages provided. Each of the 67 cases was submitted five times to each of the three models under each of the four prompting conditions, yielding a total of 4,020 individual LLM queries (67 cases × 3 models × 4 conditions × 5 runs). Between each submission, a new conversation session was initiated to prevent cross-contamination of context across runs and conditions. The five runs per case-by-condition cell were performed sequentially within the same session day for each model, and the order in which the four conditions were administered was held constant across cases.

# M4. Outcome Classification and Majority Voting

## M4.1 Three- and four-level outcomes

Each free-text model response was normalised by conversion to lower case and removal of surrounding whitespace before being mapped to a categorical outcome. The primary three-level outcome classified each response as NOT TREAT, TREAT (encompassing both endovascular coiling and microsurgical clipping) or HEDGE. For analyses of treatment modality, a four-level outcome was used that preserved the distinction between TREAT (COIL) and TREAT (CLIP) alongside NOT TREAT and HEDGE. The MDT reference standard was binary at the treat-versus-observe level (NOT TREAT or TREAT) and three-level at the modality level (NOT TREAT, TREAT (COIL), TREAT (CLIP)), as the MDT did not return hedged decisions.

## M4.2 The HEDGE category

The HEDGE category was operationalised to capture all non-committal model outputs that did not constitute a single, definitive treatment recommendation. Specifically, a response was classified as HEDGE if it fell into one of three patterns: (i) an explicit refusal to commit to a recommendation, in which the model declined to provide a binary treat-versus-observe decision and instead offered general information, options for the patient to consider, or a recommendation to consult a specialist without itself making a choice; (ii) a mixed response, in which the model recommended both treatment and observation as equally reasonable alternatives without selecting between them; or (iii) a response in which the model recommended treatment in principle but declined to specify a modality, returning neither COIL nor CLIP. All HEDGE classifications were performed deterministically from the structured output fields of the model response, and no case required subjective interpretation.

## M4.3 Majority-vote derivation

For each of the 804 model-case-condition cells (67 cases × 3 models × 4 conditions), the recommendation was derived as the majority vote across the five independent runs. Where the five runs returned three or more responses in the same outcome category, that category was assigned as the cell-level recommendation. Where no single outcome achieved a majority across the five runs, the cell was assigned to HEDGE on the principle that the model had failed to converge on a single definitive recommendation under that condition; in practice this occurred in fewer than 2% of cells across the dataset. The cell-level majority-vote outcome is the primary unit of analysis throughout the manuscript and the supplement.

# M5. Statistical Analysis

## M5.1 Software, significance threshold and reporting framework

All analyses were performed in R (version 4.5), with statistical significance set at α = 0.05 (two-sided). Consistent with our prior MDT-benchmarking study, no adjustment for multiple comparisons was applied across the family of model-by-condition contrasts, and all reported p-values are therefore presented as descriptive rather than confirmatory. Cohen’s κ was computed using the psych package (function psych::cohen.kappa); McNemar’s exact test was computed using the exact2x2 package (function exact2x2::mcnemar.exact); exact binomial tests of symmetry were computed using base R (function stats::binom.test); and Bowker’s symmetry test was implemented by Monte Carlo permutation as described below.

## M5.2 Concordance and directional preference relative to MDT

Within each model-by-condition cell, the recommendation distribution was first summarised as counts and percentages across the four outcome levels (NOT TREAT, TREAT (COIL), TREAT (CLIP) and HEDGE). Agreement between each model’s majority-vote recommendation and the binary MDT decision (TREAT or NOT TREAT) was then quantified on the analytic subset of cases in which the model returned a definitive recommendation, with HEDGE cases excluded from this analysis as they did not constitute a binary recommendation. For each cell, three quantities were reported: raw concordance as a percentage; Cohen’s κ with its asymptotic 95% confidence interval; and McNemar’s exact test on the 2 × 2 table of discordant pairs (LLM = TREAT / MDT = NOT TREAT versus LLM = NOT TREAT / MDT = TREAT). McNemar’s exact test assessed whether the discordances between model and MDT were directionally asymmetric, and a directional preference (over-treatment or under-treatment) was declared only where McNemar’s exact p was below 0.05. Cohen’s κ was interpreted using the Landis and Koch framework (<0.00 poor; 0.00–0.20 slight; 0.21–0.40 fair; 0.41–0.60 moderate; 0.61–0.80 substantial; 0.81–1.00 almost perfect).

## M5.3 Per-case migration analysis: paired contrasts and exact binomial symmetry

To characterise how individual cases migrated between successive prompting conditions, three pre-specified paired contrasts were analysed within each model: 3P → 1PN (the effect of perspective shift), 1PN → 1PAT (the effect of treatment-directed anxiety) and 1PN → 1PANT (the effect of rupture-directed anxiety). For each contrast and each model, four directional shift types were tabulated: TREAT → NOT TREAT, TREAT → HEDGE, NOT TREAT → HEDGE and CLIP → COIL (the last restricted to cases that remained TREAT in both conditions). For each shift type the forward count (cases moving in the column-header direction) and the reverse count (cases moving in the opposite direction) were recorded and tested for symmetry using the exact binomial test (stats::binom.test) under the null hypothesis that forward and reverse shifts were equally likely. Where the combined number of discordant cases (forward + reverse) was fewer than five, the p-value was suppressed and the cell reported as having insufficient n, on the basis that exact binomial power is inadequate at such counts and that any reported p-value would risk being interpreted with greater confidence than the underlying evidence supports.

## M5.4 Modality transition matrices and Bowker’s symmetry test

The complete pattern of modality transitions for each paired contrast was additionally displayed as a 3 × 3 paired transition matrix on the three-modality outcome (NOT TREAT, TREAT (COIL), TREAT (CLIP)), with cases hedged in either condition excluded from the matrix. The diagonal cells of the matrix represented cases whose outcome was identical across the two conditions, and the off-diagonal cells represented directional shifts. The symmetry of each matrix was assessed using Bowker’s test of symmetry, in which the null hypothesis is that the matrix is symmetric about the diagonal (i.e., that shifts in any given direction are matched by an equal number of shifts in the reverse direction). Because the cell counts within several of the matrices were small and the asymptotic χ² approximation to Bowker’s statistic is unreliable under such conditions, the p-value for each matrix was estimated by 10,000-replicate Monte Carlo permutation under the exchangeability null, using a fixed random seed (42) to ensure reproducibility. Significant Bowker p-values indicated that the framing manipulation drove predominantly unidirectional shifts in the modality outcome rather than a balanced exchange of cases between modalities.

## M5.5 Clinical direction of induced shifts (Figure 3 method)

To establish whether the shifts elicited by framing carried each model toward or away from MDT consensus, every per-case shift was classified by its direction relative to the MDT decision under a strict HEDGE-exclusion rule on the three-modality outcome {NOT TREAT, TREAT (COIL), TREAT (CLIP)}.

For each of the three pre-specified paired contrasts (3P → 1PN, 1PN → 1PAT and 1PN → 1PANT) and for each of the three models, the 67 cases were classified on the three-modality outcome under both conditions of the contrast. Cases whose majority-vote outcome was HEDGE in either of the two conditions were excluded from that contrast (reported as “n HEDGE excluded”), and cases whose three-modality outcome was identical in the two conditions were also excluded (reported as “n unchanged”). The remaining “changed” cases were classified by their outcome in condition B: a case was classified as progressive if its three-modality outcome in condition B exactly matched the MDT modality for that case, and as regressive otherwise. The regressive category therefore encompassed both wrong-modality shifts within the TREAT axis (e.g., a case shifted from TREAT (COIL) to TREAT (CLIP) where the MDT had recommended TREAT (COIL)) and wrong-axis shifts between TREAT and NOT TREAT (e.g., a case shifted from NOT TREAT to TREAT (COIL) where the MDT had recommended NOT TREAT). Counts were reported descriptively as absolute case counts, with the HEDGE-exclusion count and the unchanged count reported alongside to make the selection mechanism transparent to the reader and to ensure that the denominator of 67 cases per model-by-contrast cell could be reconciled from the supplementary annotations. No inferential tests were applied to this analysis, as the purpose of the classification was to characterise the directionality of induced shifts rather than to test a hypothesis about their magnitude. The full direction-classification output is presented as Figure 3 of the main manuscript.

# M6. Linguistic Analysis of LLM Response Text Under Anxiety Conditions

## M6.1 Rationale

The primary analyses (§M5) characterised what each model recommended in categorical form (NOT TREAT, TREAT (COIL), TREAT (CLIP) or HEDGE) under the four prompting conditions. They did not characterise how the recommendation was delivered. Because patient-facing clinical text carries persuasive weight independent of its categorical content, and because qualitative inspection of the categorical-HEDGE results raised the possibility that the rhetorical surface of the responses was shifting in patterns that the categorical analysis could not detect, we conducted a pre-specified linguistic analysis of the full free-text response under the two anxiety conditions (1PAT and 1PANT) to characterise the rhetorical features that accompany the categorical recommendations.

The analysis was designed to (i) quantify the form (length, empathy, disclaimers, validation, hedging language, confidence markers) of every response under the two anxiety conditions; (ii) characterise how the wording profile shifts within each model between 1PAT and 1PANT, on paired within-case data; and (iii) characterise how the wording profile differs across the three models within each condition. The analysis is reported descriptively, consistent with the presentation adopted in the main manuscript.

## M6.2 Data

The linguistic analysis used the full free-text response from every one of the five independent runs per case under the two anxiety conditions, yielding a total of 2,010 individual responses (3 models × 2 conditions × 67 cases × 5 runs). The two neutral conditions (3P and 1PN) were not analysed at the per-response level because the full free-text response under those conditions was not retained at the per-response level in the source data; only the categorical majority-vote outcome was preserved. The linguistic analysis is therefore restricted to the two anxiety conditions, and the categorical contrast across the four conditions remains intact and is reported in §M5.

## M6.3 Features extracted

Twelve linguistic features were computed for each of the 2,010 responses, comprising four continuous features and eight binary indicator features. The four continuous features were word count (whitespace-delimited tokens), sentence count (text split on terminal punctuation followed by whitespace), linguistic-hedging density (count of hedging tokens per 100 words), and confidence-marker density (count of confident tokens per 100 words). The eight binary features were: empathy opener (any empathy-lexicon hit within the first 200 characters of the response); anxiety word in opener (any token matching the regular expression \\banxi\\w+\\b within the first 200 characters); empathy mention anywhere (any empathy-lexicon hit anywhere in the response); anxiety mention anywhere; clinical disclaimer anywhere (any disclaimer-lexicon hit); patient-validation phrase anywhere; guideline or literature reference anywhere; and categorical recommendation appearing within the first 50 characters of the response.

All lexicon matches were performed case-insensitively against the full response text, with regular expressions specified prospectively rather than tuned to the data. The seven lexicons used — reproduced verbatim from the analysis script — are listed in compact form below; the full regular expressions are preserved in the analysis code archive associated with the manuscript.

```
EMPATHY: 'I (completely) understand', 'I hear you', 'I can imagine', 'completely natural/valid/
    reasonable', 'I empathise/empathize', 'it's natural/completely/reasonable/understandable',
    'incredibly stressful/scary/frightening/difficult', 'your concerns/fears/anxiety are valid/
    understandable/natural', 'feeling so/very anxious', 'how anxious/stressful/scary'

ANXIETY_WORD:  \banxi\w+\b   (matches anxious, anxiety, anxieties, etc.)

DISCLAIMER: 'I'm not (a/your) doctor/neurosurgeon/specialist/physician', 'I am an AI/artificial
    intelligence', 'not a substitute', 'please consult/discuss this with', 'must/should be
    made/discussed/confirmed/reviewed with your', 'review your actual imaging', 'ultimately
    the decision belongs to you and your', 'this is not medical/professional advice',
    'speak to/with your neuro\w+/treating/team/specialist'

HEDGE_LANG: 'might', 'could', 'may', 'perhaps', 'possibly', 'consider', 'considering', 'lean',
    'leaning', 'suggest', 'suggests', 'potentially', 'likely', 'probably', 'reasonable',
    'nuance', 'nuanced', 'close call', 'warrant(s)', 'tend(s) to', 'sometimes', 'in many/some
    cases', 'on balance', 'generally', 'typically'

CONFIDENT: 'clearly', 'definitely', 'definitive', 'strongly', 'the right choice/approach/answer',
    'must', 'should', 'recommendation:', 'strong (clinical) consensus/evidence/recommendation',
    'unequivocal', 'without question', 'firmly', '(the) best/optimal/correct/clear choice/
    management/approach/recommendation'

VALIDATION: 'you're right', 'your concern(s) is/are valid', 'valid concern', 'legitimate concern',
    'completely valid/understandable/reasonable', 'makes (complete/total) sense', 'entirely
    valid/natural/reasonable'

GUIDELINE_REF: 'ISUIA', 'UCAS(-Japan)', 'PHASES', 'UIATS', 'AHA(/ASA)', 'ESO (guidelines)',
    '(clinical/published) guidelines', 'meta-analys(i/e)s', 'systematic review', 'current
    guidance/evidence/literature'
```

## M6.4 Aggregation and reporting

For each of the six LLM-by-condition cells, per-response features were aggregated to two distinct levels of analysis. Cell-level aggregates (mean, standard deviation and asymptotic 95% confidence intervals) were computed across all 335 responses per cell, using the normal approximation `mean ± 1.96 × SE` throughout. Case-level means were computed by collapsing the five responses per case to a single value per case per condition; the case-level mean structure preserves the within-case pairing of the design and avoids overstating precision through treatment of dependent within-case responses as independent.

# M7. Compute Resources

All LLM queries were performed via consumer web interfaces at no direct computational cost to the investigators. No local GPU, cloud compute or API billing was involved. Approximate inference time per query ranged from 5 to 90 seconds depending on the model and the latency of the extended-thinking mode where enabled, with the longest inference times typically observed under Claude Opus 4.6 Extended and Gemini 3 Pro Thinking. The cumulative wall-clock time across all 4,020 individual LLM queries was approximately 60–80 hours of inference, distributed across the three models and four prompting conditions and performed over a three-week period.

**Supplementary Table 1: Breakdown of HEDGE category**

| **LLM** | **Condition** | **NOT TREAT** | **TREAT (with clear modality recommended)** | **HEDGE category** | | **Total HEDGE** | **% Treating but without clear modality of hedges** |  |
| --- | --- | --- | --- | --- | --- | --- | --- | --- |
|  |  |  |  | **TREAT (unclear modality)** | **No definitive recommendation** |  |  | **% Completely not definitive** |
| Gemini | 3P | 35 | 32 | 0 | 0 | 0 | — | — |
|  | 1PN | 42 | 21 | 2 | 2 | 4 | 50% | 50% |
|  | 1PAT | 42 | 24 | 1 | 0 | 1 | 100% | 0% |
|  | 1PANT | 41 | 20 | 3 | 3 | 6 | 50% | 50% |
| Claude | 3P | 44 | 23 | 0 | 0 | 0 | — | — |
|  | 1PN | 39 | 24 | 3 | 1 | 4 | 75% | 25% |
|  | 1PAT | 39 | 15 | 8 | 5 | 13 | 62% | 38% |
|  | 1PANT | 41 | 19 | 6 | 1 | 7 | 86% | 14% |
| GPT | 3P | 38 | 29 | 0 | 0 | 0 | — | — |
|  | 1PN | 38 | 29 | 0 | 0 | 0 | — | — |
|  | 1PAT | 37 | 29 | 1 | 0 | 1 | 100% | 0% |
|  | 1PANT | 35 | 32 | 0 | 0 | 0 | — | — |
