# Supplementary material for: The Influence of Patient Persona and Affective Framing on Management Recommendations of ChatGPT, Gemini and Claude for Unruptured Intracranial Aneurysms: A Comparative Benchmarking Study
Source: Neurosurg Rev. 2026 Aug 3;49(1):508. doi: 10.1007/s10143-026-04419-2 (PMC13433641; doi:10.1007/s10143-026-04419-2)
Supplement: Supplementary file 2 [file 10143_2026_4419_MOESM2_ESM.docx]

**TRIPOD-LLM Reporting Checklist**

*Completed for: The Influence of Patient Persona and Affective Framing on Management Recommendations of ChatGPT, Gemini and Claude for Unruptured Intracranial Aneurysms: A Comparative Benchmarking Study*

*Reporting guideline: Gallifant J, Afshar M, Ameen S, et al. The TRIPOD-LLM reporting guideline for studies using large language models. Nature Medicine 2025;31(1):60–69. doi:10.1038/s41591-024-03425-5.*

## Study classification

Under the TRIPOD-LLM modular framework, the present study is classified as follows. This classification determines which items of the full 19-item / 50-subitem checklist apply.

| **Module** | **Selection and rationale** |
| --- | --- |
| **Research design** | LLM evaluation (E). The study evaluates three existing, publicly available frontier LLMs (Claude Opus 4.6, ChatGPT-5.4, Gemini 3 Pro Thinking) against an institutional MDT reference standard. No new LLM was developed, no instruction tuning or alignment modelling was performed, and no model was deployed within a live clinical workflow. The study is therefore not classified under de novo LLM development (D), LLM methods (M), or LLM evaluation in healthcare settings (H). |
| **LLM task** | Classification (C). Each LLM output was mapped to one of three pre-specified categorical recommendations (NOT TREAT, TREAT (COIL), TREAT (CLIP)) with HEDGE as a non-committal class. Free-text rationales accompanied each recommendation but were not formally graded as generative outputs, and the study endpoint is therefore the categorical recommendation rather than the natural-language explanation. |

## Main checklist

| **Item** | **Checklist item (paraphrased)** | **Where addressed** | **Status** |
| --- | --- | --- | --- |
| **Title** | | | |
| **1** | Identify the study as developing, fine-tuning and/or evaluating an LLM, specifying the task, the target population and the outcome. | Title page — ‘The Influence of Patient Persona and Affective Framing on Management Recommendations of ChatGPT, Gemini and Claude for Unruptured Intracranial Aneurysms: A Comparative Benchmarking Study’. Identifies the study as an evaluation of three named LLMs (task: management recommendation; population: UIA patients; outcome: LLM categorical recommendation). | Reported |
| **Abstract** | | | |
| **2** | TRIPOD-LLM for abstracts followed | Abstract | Reported |
| **Introduction — Background** | | | |
| **3a** | Explain the healthcare context and rationale for evaluating the LLM, with references to existing approaches and models. | Introduction, paragraphs 1–2. Establishes patient-facing use of LLMs prior to specialist review for UIA management, the equipoise nature of the management decision, and the existing literature documenting fair-to-moderate LLM–expert agreement. | Reported |
| **3b** | Describe the target population and the intended use of the LLM in the context of the care pathway (E,H / All). | Introduction, paragraph 1. Target population: adult UIA patients consulting frontier LLMs prior to specialist review. Intended use being evaluated: pre-consultation informational support and patient-facing recommendation generation. | Reported |
| **Introduction — Objectives** | | | |
| **4** | Specify the study objectives, including whether the study describes development, fine-tuning or validation of an LLM. | Introduction, paragraph 3. Primary objective: quantify how patient persona and affective framing alter UIA management recommendations across three LLMs. Secondary objective: characterise the direction of induced shifts relative to MDT consensus. | Reported |
| **Methods — Data** | | | |
| **5a** | Describe sources of data separately for training, tuning, and/or evaluation datasets and the rationale. | Materials and Methods §Study design and case cohort; Supplement M1.1. Evaluation dataset: 67 consecutive UIA cases discussed at the institutional neurovascular MDT, January–December 2025. No training or tuning data (LLM evaluation only). | Reported |
| **5b** | Describe relevant data points and provide a quantitative and qualitative description of their distribution. | Supplement M1.2 and Table M1; main manuscript Table 1 (baseline cohort characteristics). 27 clinical variables across five domains. | Reported |
| **5c** | Specifically state the date of the oldest and newest item of text used in the development and evaluation datasets. | Materials and Methods §Study design and case cohort; Supplement M1.1. Evaluation cases drawn from MDT meetings between January and December 2025. Prompts submitted to LLMs between March and April 2026 (Supplement M3.1). | Reported |
| **5d** | Describe any data preprocessing and quality checking, including whether this was similar across text corpora, institutions and sociodemographic groups. | Supplement M1.2. Two-reviewer extraction (MF, AN) into a bespoke template with FM adjudication; single-institution cohort. | Reported |
| **5e** | Describe how missing and imbalanced data were handled and provide reasons for omitting any data. | Supplement M1.2. Missing variables populated with ‘Not documented’ or ‘assumed no’ to prevent LLM inference of unstated information. | Reported |
| **Methods — Analytical methods** | | | |
| **6a** | Report the LLM name, version and last date of training. | Materials and Methods §Large language models and prompt administration; Supplement M3.1 and Table M2. Models named with version: Claude Opus 4.6, ChatGPT-5.4, Gemini 3 Pro Thinking. Inference dates: March–April 2026. The training-data cutoff dates are not publicly disclosed by Anthropic, OpenAI or Google for the consumer-web-interface versions of these models, and this is noted as a constraint on temporal reproducibility in the manuscript Limitations. | Reported |
| **6b** | Report details of the LLM development process (M,D / All). | Not applicable. Study is LLM evaluation (E), not development (D) or methods (M); no LLM development was performed. | *NA* |
| **6c** | Report details of how text was generated using the LLM, including prompt engineering and inference settings (M,D,E / All). | Supplement M2 (prompt design, all four prompting conditions reproduced verbatim in M2.3); Supplement M3.2 and Table M2 (inference settings: web-interface defaults, extended-thinking enabled where available, no system instructions, no RAG, web browsing and memory disabled, no user-configurable temperature/top-p/seed). | Reported |
| **6d** | Specify the initial and postprocessed output of the LLM (e.g., probabilities, classification, unstructured text). | Supplement M4.1–4.2. Initial output: free-text response containing a categorical recommendation, modality and rationale. Postprocessed output: categorical mapping to NOT TREAT, TREAT (COIL), TREAT (CLIP) or HEDGE. | Reported |
| **6e** | Provide details and rationale for any classification and, if applicable, how probabilities were determined and thresholds identified (All / C, OF). | Supplement M4.1–4.3. Three-level outcome with HEDGE as a fourth category; majority-vote derivation across five independent runs (3-of-5 majority threshold); HEDGE assigned where no majority emerged. | Reported |
| **Methods — LLM output** | | | |
| **7a** | Include metrics that capture the quality of generative outputs (consistency, relevance, accuracy, errors). Applicable to QA, IR, DG, SS, MT tasks. | Not applicable. The study task is classification (C); the free-text rationales accompanying each categorical recommendation were not formally graded as generative outputs and no generative-quality metric was applied. Run-to-run consistency across the five independent runs per cell is captured by the majority-vote derivation (Supplement M4.3). | *NA* |
| **7b** | Report the outcome metrics’ relevance to the downstream task at deployment and, where applicable, correlation of metric to human evaluation of the text (E,H / All). | Materials and Methods §Outcomes; §Statistical analysis. Metrics chosen for their direct interpretability against MDT decision-making: raw concordance, Cohen’s κ, McNemar’s exact for directional preference, exact binomial and Bowker’s tests for paired-condition migration. | Reported |
| **7c** | Clearly define the outcome, how the LLM predictions were calculated, the date of inference for closed-source LLMs and the evaluation metrics (E,H / All). | Materials and Methods §Outcome classification and §Statistical analysis; Supplement M3.1 (inference dates March–April 2026); Supplement M5 (all evaluation metrics with software packages). | Reported |
| **7d** | If outcome assessment requires subjective interpretation, describe assessor qualifications, instructions and inter-assessor agreement. | Supplement M4.1–4.2. Outcome classification was deterministic from structured fields of the LLM output and did not require subjective interpretation; inter-assessor agreement therefore does not apply at the outcome-classification step. | Reported |
| **7e** | Specify how performance was compared to other LLMs, humans and other benchmarks or standards. | Materials and Methods §Outcomes and Results §Agreement with MDT. Performance compared head-to-head between three LLMs and against the institutional MDT consensus as the human reference standard. | Reported |
| **Methods — Annotation** | | | |
| **8a** | If annotation was done, report how the text was labeled, including specific annotation guidelines with examples. | Supplement M4.1–4.2. Mapping rules for the three-level and four-level outcomes are stated, with three patterns operationalising the HEDGE category. | Reported |
| **8b** | Report how many annotators labeled the dataset(s), inter-annotator agreement, and the proportion of data annotated in duplicate. | Supplement M1.2 (case-data extraction: two reviewers, FM adjudicator); Supplement M4.1 (LLM-output classification was deterministic, single-pass, no inter-annotator agreement applicable). | Reported |
| **8c** | Provide information on the background and experience of the annotators or the characteristics of any models involved in labeling. | Author affiliations on title page (MF, AN — medical doctors/research fellows; FM adjudicator). No model-based labelling was used. | Reported |
| **Methods — Prompting** | | | |
| **9a** | If research involved prompting LLMs, provide details on the processes used during prompt design, curation and selection. | Supplement M2.1. Three iterations of pilot testing on non-study cases, with revisions targeting variable completeness, output-format enforceability, and run-to-run consistency. No alternative prompt architectures (chain-of-thought, few-shot, role-play) were formally tested. | Reported |
| **9b** | If research involved prompting LLMs, report what data were used to develop the prompts. | Supplement M2.1. Pilot data: non-study UIA cases from outside the January–December 2025 analytic period. | Reported |
| **Methods — Summarization (All / SS)** | | | |
| **10** | Describe any preprocessing of the data before summarization. | Not applicable. Study task is classification (C), not summarisation (SS). | *NA* |
| **Methods — Instruction tuning / alignment (M, D / All)** | | | |
| **11** | If instruction tuning/alignment strategies were used, report the instructions, data, interface and population characteristics. | Not applicable. Study is LLM evaluation (E); no instruction tuning or alignment modelling was performed by the investigators. | *NA* |
| **Methods — Compute (M, D, E / All)** | | | |
| **12** | Report compute, or proxies thereof (time, cost, hardware, FLOPS). | Supplement M6. Inference performed via consumer web interfaces at no direct computational cost; per-query latency 10–90 seconds; cumulative wall-clock approximately 60–80 hours across 4,020 queries over a four-week period. | Reported |
| **Methods — Ethical approval** | | | |
| **13** | Name the IRB or ethics committee that approved the study and describe consent or its waiver. | Manuscript §Statements and Declarations → Ethics approval; Materials and Methods §Study design and case cohort. Institutional review board approval RES-25-0000-885Q; individual informed consent waived owing to the retrospective use of fully de-identified data, with no identifiable patient information submitted to any LLM at any point. | Reported |
| **Open science** | | | |
| **14a** | Give the source of funding and the role of the funders. | Manuscript §Statements and Declarations → Funding. The authors declare that no funds, grants, or other support were received during the preparation of this manuscript. | Reported |
| **14b** | Declare any conflicts of interest and financial disclosures for all authors. | Manuscript §Statements and Declarations → Competing Interests. The authors have no relevant financial or non-financial interests to disclose. | Reported |
| **14c** | Indicate where the study protocol can be accessed (H / All). | Not applicable. Study is classified E (LLM evaluation), not H (LLM evaluation in healthcare settings); protocol publication not required by item. | *NA* |
| **14d** | Provide registration information for the study (H / All). | Manuscript §Statements and Declarations → Registration. The study is registered on the Open Science Framework: [**10.17605/OSF.IO/HCU6D**](https://doi.org/10.17605/OSF.IO/HCU6D) Although item 14d is not formally required under the LLM-evaluation (E) research-design classification, the registration is reported voluntarily for transparency. | Reported (voluntary) |
| **14e** | Provide details of the availability of the study data. | Manuscript §Statements and Declarations → Data availability. De-identified patient data and LLM output data are available from the corresponding author upon reasonable request, subject to institutional ethics approval. | Reported |
| **14f** | Provide details of the availability of the code to reproduce the study results. | Manuscript §Statements and Declarations → Code availability. The R scripts used for statistical analysis are available from the corresponding author upon reasonable request via email. | Reported |
| **Patient and public involvement** | | | |
| **15** | Provide details of any patient and public involvement (H / All). | Not applicable. Study is classified E, not H. No patient or public involvement in design, conduct, reporting, interpretation or dissemination. | *NA* |
| **Results — Participants (E, H / All)** | | | |
| **16a** | Describe the flow of text/EHR/patient data through the study, including documents/questions/participants with and without the outcome. | Materials and Methods §Study design and case cohort; Results paragraph 1. 67 cases included from the January–December 2025 MDT cohort, harbouring 99 aneurysms; flow consistent with the analytic cohort denominator of 67 cases per model-by-condition cell. | Reported |
| **16b** | Report the characteristics overall and for each data source / setting / split. | Results paragraph 1; main-manuscript Table 1 (baseline characteristics). Single-institution dataset; no separate development/evaluation split (evaluation-only study). | Reported |
| **16c** | For LLM evaluation including clinical outcomes, compare distributions of important clinical variables between development and evaluation data. | Not applicable. Evaluation-only study with a single cohort; no development/evaluation split exists for comparison. | *NA* |
| **16d** | Specify the number of participants and outcome events in each analysis (development, hyperparameter tuning, evaluation). | Materials and Methods §Study design and case cohort; Results paragraph 1; main-manuscript Tables 1–3. 67 cases, 18 MDT TREAT decisions (10 COIL, 8 CLIP), 49 NOT TREAT. | Reported |
| **Results — Performance** | | | |
| **17** | Report LLM performance according to prespecified metrics and/or human evaluation. | Results §Treatment recommendations across prompting conditions (Table 1); §Agreement with the multidisciplinary team gold standard (Table 2); §Per-case stability and directional shifts across paired conditions (Table 3, Figure 2); §Clinical direction of induced shifts (Figure 3). | Reported |
| **Results — LLM updating** | | | |
| **18** | If applicable, report the results from any LLM updating, including the updated LLM and subsequent performance. | Not applicable. No LLM updating was performed within this study; all three models were evaluated at fixed published versions accessed during March–April 2026. | *NA* |
| **Discussion — Interpretation** | | | |
| **19a** | Give an overall interpretation of the main results, including issues of fairness in the context of the objectives and previous studies. | Discussion §Findings. Three alignment-architecture-specific sycophantic phenotypes characterised across Claude, Gemini and ChatGPT, situated within the prior sycophancy literature (Kim et al., Chen et al., Hadjiathanasiou et al.) and the broader RLHF and Constitutional AI alignment literature. | Reported |
| **19b** | Discuss any limitations of the study and their effects on biases, statistical uncertainty and generalisability. | Discussion §Limitations. Five limitations enumerated: single-turn evaluation; researcher-constructed personas; model-version snapshot; black-box mechanism inference; single-centre MDT reference standard with no downstream outcome validation. | Reported |
| **Discussion — Usability of the LLM in context (E, H / All)** | | | |
| **19c** | Describe known challenges in using data for the specified task and domain context (representation, missingness, harmonisation, bias). | Discussion §Limitations (researcher-constructed personas, single-centre MDT, model-snapshot limitations); Discussion §Findings (training-corpus asymmetries favouring intervention). | Reported |
| **19d** | Define the intended use for the implementation under evaluation, including the intended input, end-user and level of autonomy/human oversight. | Introduction paragraph 1; Discussion §Clinician implications. Intended use of the LLM under evaluation: patient-facing pre-consultation informational support; intended end-user: patient; required level of human oversight: full MDT and clinician review prior to any clinical decision. | Reported |
| **19e** | If applicable, describe how poor-quality or unavailable input data should be assessed and handled when implementing the LLM. | Supplement M1.2 (handling of missing variables); Discussion §Limitations (researcher-constructed personas not equivalent to real patient inputs). | Reported |
| **19f** | If applicable, specify whether users will be required to interact in the handling of the input data or use of the LLM, and the expertise required of users. | Discussion §Clinician implications (algorithmic literacy as a formal component of pre-consultation counselling; patient end-users do not require technical expertise but clinicians require it to interpret AI-shaped patient expectations). | Reported |
| **19g** | Discuss any next steps for future research, with a specific view of the applicability and generalisability of the LLM. | Discussion §Future Directions. Five threads enumerated: multi-turn conversational benchmarking; prospective real-world patient evaluations; expanded persona space; multi-institutional benchmarking; anti-sycophancy interventions. | Reported |

*.*
